# RC2S: A Cognitive Remediation Program to Improve Social Cognition in Schizophrenia and Related Disorders

**DOI:** 10.3389/fnhum.2014.00400

**Published:** 2014-06-13

**Authors:** Elodie Peyroux, Nicolas Franck

**Affiliations:** ^1^Rehabilitation Department (CL3R), Le Vinatier Hospital, Lyon, France; ^2^UMR 5229, Center of Cognitive Neurosciences, CNRS, Bron, France; ^3^University of Lyon, Lyon, France

**Keywords:** social cognition, schizophrenia and related disorders, cognitive remediation, simulation techniques, social functioning

## Abstract

In people with psychiatric disorders, particularly those suffering from schizophrenia and related illnesses, pronounced difficulties in social interactions are a key manifestation. These difficulties can be partly explained by impairments in social cognition, defined as the ability to understand oneself and others in the social world, which includes abilities such as emotion recognition, theory of mind (ToM), attributional style, and social perception and knowledge. The impact of several kinds of interventions on social cognition has been studied recently. The best outcomes in the area of social cognition in schizophrenia are those obtained by way of cognitive remediation programs. New strategies and programs in this line are currently being developed, such as RC2S (cognitive remediation of social cognition) in Lyon, France. Considering that the social cognitive deficits experienced by patients with schizophrenia are very diverse, and that the main objective of social cognitive remediation programs is to improve patients’ functioning in their daily social life, RC2S was developed as an individualized and flexible program that allows patients to practice social interaction in a realistic environment through the use of virtual reality techniques. In the RC2S program, the patient’s goal is to assist a character named Tom in various social situations. The underlying idea for the patient is to acquire cognitive strategies for analyzing social context and emotional information in order to understand other characters’ mental states and to help Tom manage his social interactions. In this paper, we begin by presenting some data regarding the social cognitive impairments found in schizophrenia and related disorders, and we describe how these deficits are targeted by social cognitive remediation. Then we present the RC2S program and discuss the advantages of computer-based simulation to improve social cognition and social functioning in people with psychiatric disorders.

## Introduction

Adequate social functioning is the consequence of a person’s ability to interact appropriately and effectively in the social world (Hooley, 2010). In people with psychiatric disorders, particularly those suffering from schizophrenia and related illnesses, pronounced difficulties in social interactions are a key manifestation (Bellack et al., 1990). Deterioration of social relations was recognized a century ago in the earliest clinical descriptions of the schizophrenia, and still remains a hallmark of the disorder at all of its stages. Individuals with psychiatric disorders are often socially isolated, unemployed, unable to manage money, and generally lack the skills to live independently. Moreover, these impairments and disabilities are often not improved by psychotropic medication. Poor social-skills may promote relapses and have a negative impact on interpersonal support, social affiliation, and quality of life (Kopelowicz et al., 2006). Interpersonal and relational problems exacerbate symptoms, participate in psychological suffering, and slow down the rehabilitation process (Prouteau, 2011). Impaired social functioning cannot be accounted for solely the symptoms of the disorder or the effects of medication and hospitalization (Hooley, 2010). It may in fact be an early marker of schizophrenia.

Interpersonal difficulties can be partly explained by impaired social cognition, defined as the ability to understand oneself and others in the social world (Penn et al., 2008) or to construct mental representations about others and oneself, and about one’s relationships to others (Brothers, 1990). Currently, a consistent and significant scientific body of literature attests to dysfunctional social cognition in schizophrenia (Penn et al., 1997; Green et al., 2005, 2008). According to the American Psychiatric Association (2000), impaired social functioning is one of the hallmark characteristics of schizophrenia and also one of the most important unmet treatment needs for people suffering from this mental disease (Kern et al., 2009).

## Cognitive Impairments in Schizophrenia

Cognitive impairments are a core feature of schizophrenia that is strongly associated with functioning in areas such as work, social relationships, and independent living (McGurk et al., 2007). The cognitive deficits of people with schizophrenia and related disorders are found in the domains of neurocognition and social cognition. Their impaired neurocognitive functions are now well documented, and the most pronounced are in the areas of attention, verbal memory, and executive functioning (Medalia and Choi, 2009). To address these critical problems facing people with schizophrenia, a range of cognitive remediation programs have been developed and evaluated over the past 40 years. The principle underlying this therapeutic approach is the enhancement of patients’ cognitive resources in view of improving their cognitive functions, and indirectly, the functional disabilities that impinge upon their daily lives (Demily and Franck, 2008). In the past few years, many authors have studied the links between cognition and functional outcome in schizophrenia. Despite the significant association between neurocognition and functional impairment, correlations with composite measures of neurocognition are only moderately intense, however, explaining a modest part of the variance in functional outcomes.

These results have prompted the search for other factors likely to enhance our understanding of the relationships between cognition and functional deficits (Schmidt et al., 2011). The most promising mediator uncovered so far lies in the area of social cognition. Hence, the first question raised by clinicians and researchers was: are neurocognition and social cognition separate factors in schizophrenia? According to the majority of studies, social cognition and neurocognition are related but are distinct constructs in schizophrenia (Allen et al., 2007; Sergi et al., 2007a; Hoe et al., 2012). Recent models, investigating the role of social cognition have proposed that this cognitive domain may serve as a mediating link between neurocognition and community functioning (Vauth et al., 2004; Schmidt et al., 2011). This implies that neurocognitive impairments may have a negative impact on social cognition and thereby exert a negative influence on the patient’s functional state (Schmidt et al., 2011). Social cognition is actually strongly associated with social functioning and plays a key role in social and community integration, work life, and interpersonal relationships (Couture et al., 2006). Another recent meta-analysis (Fett et al., 2011) showed than social cognition has a greater impact on variance in community outcome (16%) than neurocognition does (6%). Moreover, a major part of the functional prognosis of people with schizophrenia has been found to depend on social cognition disorders (Penn et al., 1996; Kee et al., 2003).

Social cognition is not a one-dimensional construct however (van Hooren et al., 2008). In schizophrenia, three to five of the components of this cognitive domain are usually altered and have specific detrimental effects on functioning in everyday life (Green et al., 2004, 2008; Penn et al., 2008). The first component is emotional processing, which is the ability to identify and recognize emotions through facial expressions, gestures, and tone of voice. Emotional processing has been widely studied in schizophrenia, and deficits in facial and vocal emotion recognition are now well established (Edwards et al., 2002). Moreover, impairments of this component may be one of the most important factors contributing to the social isolation of people with schizophrenia (Hoekert et al., 2007). The second component is theory of mind (ToM), which is defined as “the ability to attribute mental states (beliefs, intents, desires, …) to oneself and others, and to understand that others have beliefs, intentions, and desires that are different from one’s own” (Premack and Woodruff, 1978). Impaired ToM in schizophrenia is now well documented (Sprong et al., 2007; Bora et al., 2009) and this component seems to be fundamental to proper social functioning (Couture et al., 2011). The third component, attributional style, refers to how people explain the causes of positive and negative events. Generally, the causes of an event can be attributed to oneself (human internal attribution), to others (human external attribution), or to the environment (situational external attribution). In schizophrenia, people tend to blame others for their negative life events rather than sharing the responsibilities between different sources (Favrod et al., 2013). These attributional biases seem to have a direct influence on social interaction abilities. The tendency to make stable attributions for life events has been shown to predict more frequent social contacts, a higher quality of social interaction, and better community participation (Lysaker et al., 2004). The last two components, social perception and knowledge, can be defined as decoding and interpreting social cues from others, taking the social context into account, and knowing social rules, roles, and goals. Social perception and social knowledge are closely linked to community functioning and are a necessary prerequisite for well-adapted social-skills (Green et al., 2005).

Each component’s impairment has specific detrimental effects on everyday life. For example, strong correlations have been identified between emotional processing and occupational and social status, and between ToM and behavioral adaptation. Moreover, social cognition impairments seem to have an impact on psychotic symptomatology, although the association between them remains unexplored. According to Demily and Franck (2008), social cognition deficits contribute to the onset and maintenance of symptoms. Concerning positive symptoms, social cognition impairments may contribute to the sense of insecurity in schizophrenia which leads to delusions, and notably to persecutory delusions. These social cognition disorders may also favor negative symptoms through a lack of interest in others and reduced social relationships. Social cognition therefore constitutes a central target for improving the ability of people with schizophrenia to interact socially in an appropriate way.

## Cognitive Remediation of Social Cognition

The impact on social cognition of several kinds of interventions has been studied recently. Concerning antipsychotic medication, no improvement in the area of social cognition has been highlighted, even with second-generation treatments (Sergi et al., 2007b). Other techniques like the intranasal administration of oxytocin have also been investigated, with some studies showing encouraging results such as a decrease in positive symptoms (Churchland and Winkielman, 2012) and an improvement in ToM and social perception (Pedersen et al., 2011). However, the most satisfactory results for improving social cognition in schizophrenia are those obtained by cognitive remediation (Kurtz and Richardson, 2011). Several new cognitive remediation strategies and programs are currently being developed.

**Figure 1:**
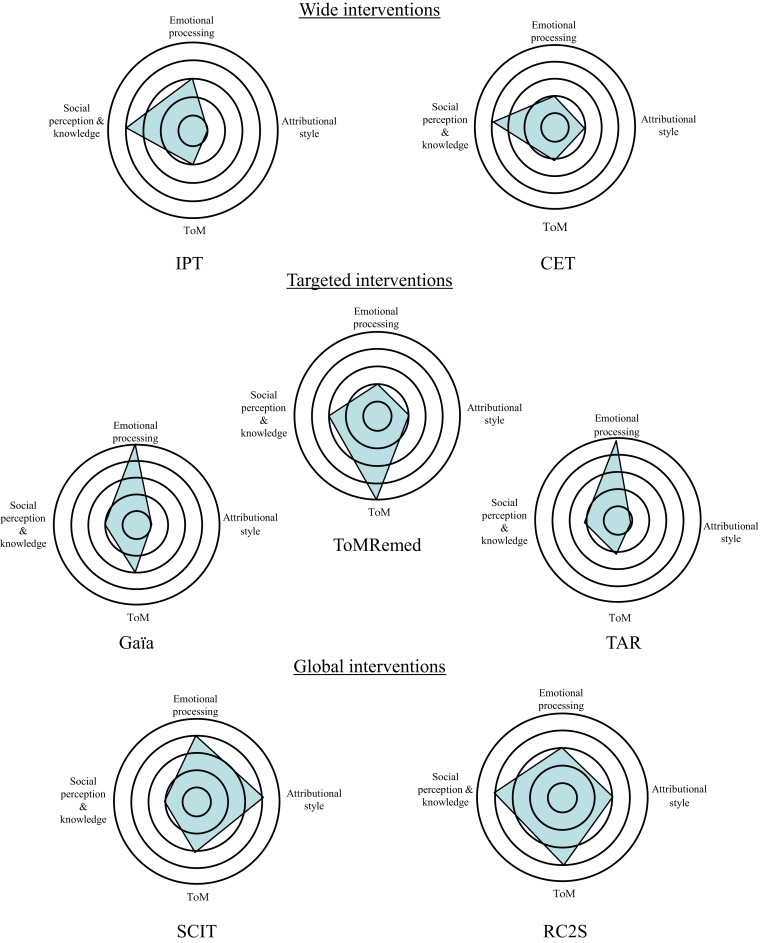
**Components of social cognition solicited according to social cognitive remediation programs (inspired by Peyroux et al., 2013)**. To determine ratings we used our own experience of the programs (IPT, Gaïa, TomRemed, SCIT, and RC2S) and descriptions of the cognitive remediation procedures described in the literature (for CET and TAR notably). Subjective quotations have been done thanks to the following scale: 1 = social cognitive domain not targeted at all by the program, 2 = marginal impact on the social cognitive process, 3 = medium impact on the social cognitive process, 4 = major impact on the social cognitive process, and 5 = specific and significant impact on the social cognitive process (central target of the program).

In the cognitive remediation of social cognition, three kinds of interventions, based on three different theoretical foundations, are available today (see Figure 1, and for a French review of cognitive remediation programs targeting social cognition in schizophrenia, see Peyroux et al., 2013). The earliest ones are called “wide interventions” and are based on the idea that the acquisition of basic cognitive skills strengthens relational competency. In this domain of intervention, two of the most well-known therapies are CET (Hogarty and Flesher, 1999; Hogarty et al., 2004) and IPT (Roder et al., 1988, 2006), a Swiss program that combines neurocognitive and social cognitive interventions with training in social-skills. Other programs called “targeted interventions” are more specific. Each one targets a specific component of social cognition such as ToM, e.g., ToMRemed a French program developed in Versailles (Bazin et al., 2010), or facial emotion recognition, e.g., TAR (Frommann et al., 2003) and Gaïa (Gaudelus and Franck, 2012) developed in Lyon and currently undergoing an efficacy study. More recently, “global interventions” have been developed. This kind of program tries to take into account all components of social cognition that are impaired in schizophrenia. In the area of global interventions, the American social cognition and interaction training (SCIT) program (Combs et al., 2007; Penn et al., 2007; Roberts and Penn, 2009) is a group-based intervention, delivered weekly over an 18-week period. SCIT is composed of three phases: emotion training (defining emotions, emotion mimicry training, and understanding paranoia), figuring out situations (distinguishing facts from guesses, jumping to conclusions, and understanding bad events), and integration (checking out guesses in real life). Its efficacy in improving social cognition components is now well established.

A recent meta-analytic study devoted to cognitive remediation of social cognition (Kurtz and Richardson, 2011), which took into account 19 studies, revealed that social cognitive training procedures have a moderate to large effect on emotion recognition, a small to moderate effect on ToM, but no effect on social perception or attributional bias. One of the most important findings in this study is that these kinds of techniques have a moderate to large effect on community functioning, which provides strong evidence for the transfert of training effects to daily social life. In another meta-analysis of social cognitive treatment for psychosis Fiszdon and Reddy (2012) reviewed nearly 50 studies. They focused on proximal social cognitive effects, the durability of those effects, and their generalization to functional measures and their conclusions are consistent with earlier findings by Kurtz and Richardson. They suggested that simple cognitive processes like emotion recognition can be definitively improved by social cognitive remediation, but that these techniques have limited success in remediating more complex, higher-order social cognitive functions. They proposed two potential reasons for these mixed results: a lack of consideration of and/or compensation for cognitive impairments, and limited opportunity to practice skills. We propose an alternative explanation, close to their second proposal: the lack of a real-world environment where patients are confronted with complex social interactions that take all components of social cognition into account. Social cognitive remediation programs today often focus on relatively simple associations and deductive reasoning about one of the components of social interaction. They use a hierarchical, step-by-step model in which each process is taught independently and thereby lacks the characteristics of the real social situation. We assume that an integrated, natural remediation program close to the real social world could be helpful in improving higher-order social cognitive functions. Thanks to technological developments this kind of program has become feasible.

## Advantages of Computer Technologies in Cognitive Remediation of Social Cognition

In the field of neurocognition, the use of specific computer programs for cognitive remediation has become very popular within the past few years (Lindenmayer et al., 2008; Franck et al., 2013; Vianin, 2013). These programs have many advantages, such as their ability to adapt the difficulty level to the specific skills of each patient, to give immediate feedback concerning performance, and to readjust the reinforcement methods used (Tomas et al., 2010). Moreover, according to a meta-analytic study on the efficacy and specificity of computer-assisted cognitive remediation in schizophrenia, prolonged multimedia stimulation is a factor that promotes neural plasticity (Grynszpan et al., 2011), a fundamental concept in cognitive remediation. Cognitive remediation appears to have an impact on the amount of gray matter over a 2-years period, in specific areas significantly related to improved cognition (Eack et al., 2010) and also seems to be related to greater efficiency of the interhemispheric transfer of information between the right and left prefrontal cortices (Penadés et al., 2013).

Very few computer programs have been used in social cognition therapy, probably because it is assumed that interacting with a machine does not involve social-skills. It seems, however, that social cognition can be learned directly via computer programs because computerized tasks often provide the opportunity to decompose and control the different processes at play in social interactions, and thus to offer a progressive training program regarding difficulty.

Other technologies, such as virtual reality or simulation techniques, are even more applicable to improve social cognition, and to promote one of the greatest challenges for cognitive remediation: the transfer of acquired abilities to everyday life following treatment and the generalization of treatment benefits to other social processes. Virtual reality can be defined as techniques founded on real-time interaction with a virtual world that helps the learner to perform specific tasks defined by computer programs (Arnaldi et al., 2006). It offers the possibility of building realistic environments in 3D. Virtual reality or simulation techniques are already being used in several fields, not only in playing and the military, but also in medical domains.

These techniques expose patients to complex, dynamic, interactive stimuli and serve to assess or remedy cognitive, behavioral, and functional impairments in tasks very close to those found in daily life. Psychiatry and neuropsychology are taking advantage of these technologies for treating specific phobias, post-traumatic stress disorder, attention deficit disorder in children, and test anxiety (for a review, see Gregg and Tarrier, 2007). For anxiety disorders, therapies using virtual reality are based on the principle of exposure, like traditional approaches. They have proven effective for treating several phobias and post-traumatic stress disorder (Riva, 2005; Gregg and Tarrier, 2007; Powers and Emmelkamp, 2008).

Although virtual reality is typically used as an exposure technique for treating the difficulties mentioned above, it has been recently applied to the study and treatment of schizophrenia. In a recent meta-analysis, Freeman (2008) counted seven applications of virtual social environments to schizophrenia: assessing symptoms, identifying symptom markers, determining predictive factors, testing putative causal factors, investigating different predictions of symptoms, searching for toxic elements in the environment, and developing treatments. In the field of assessment and treatment, virtual reality systems can provide a viable environment for individuals to interact with social avatars. According to Kim and Kim (2011), virtual reality may be one of the most promising tools for assessing social-skills because it minimizes bias resulting from traditional assessment methods.

People placed in a virtual environment are inclined to treat virtual individuals as real humans, which involves responding and interacting with them in a natural way in accordance with both the context and the affect it entails (Bailenson et al., 2003). These “humanization effects” were also reported for people with schizophrenia by Ku et al. (2006). In the study by these authors, the participant had to discuss briefly with a virtual avatar while the experimenters measured interpersonal distance between the patient and the avatar, and the patient’s verbal response time. The results supported the assumption that the avatar was perceived as a real person by the patients with schizophrenia, suggesting that communication skills could be assessed and improved by this kind of procedure.

Virtual reality and simulation techniques can also be used to generate safe and harmless environments where patients can learn social-skills without negative repercussions on their real life, such as emotional frustration or a feeling of failure. Hence, in consideration of the stigma associated with mental illness, which can be highly detrimental to rehabilitation in people with psychiatric pathologies, utilizing a virtual environment without having to fear the negative consequences of the real world may be a favorable method (Kim and Kim, 2011). For all of these reasons, a number of clinical studies have been exploring the use of virtual reality to improve conversational and communication skills.

Since 2005, an American team has launched four projects aimed at taking advantage of simulation techniques to develop programs for improving social-skills among people with autism spectrum disorders. For these patients, impaired relational commitment is one of the most debilitating symptoms (Trepagnier et al., 2005). Preliminary data obtained with this kind of technique has highlighted the benefits gained by patients in the field of social cognition. In general, studies using virtual reality or computerized techniques obtain higher scores on tests of ToM skills, more appropriate judgments about pragmatically appropriate behavior, and better recognition of facial expressions of emotion (cited in Trepagnier et al., 2011). Moreover, it seems that subjects’ real-world interactions can also be improved by these techniques. In this vein, Kandalaft et al. (2013) who studied eight adults diagnosed with high-functioning autism, found a significant increase in social cognitive measures, as well as gains in social functioning (for example, maintaining a conversation in real life, understanding other people’s point of view, and establishing relationships) after 10 sessions spread over 5 weeks of intervention.

Concerning schizophrenia, some research teams have begun to use these techniques as a therapeutic and social-skill training tool (Kim and Kim, 2011). Ku et al. (2007) designed an experiment to evaluate opinions of patients with schizophrenia about a virtual conversational skills training program. They acquired objective measures as silence-breaking times (the duration from the beginning of a silence during a conversation to talk-button pressing) and subjective measures by using questionnaires of usability, opinions, and presence. Their results indicated that patients underwent the virtual conversation program without problem and that the virtual conversation with virtual avatars could be effectively exposed to patients with schizophrenia. Currently, several clinical research teams are studying virtual reality applications in the field of social-skill training. In their clinical study, Park et al. (2011) compared training in social-skills training based on traditional role-playing to another method using virtual reality role-playing. They found that the virtual reality system was particularly good at improving conversational skills and assertiveness, and concluded that these techniques may be a useful supplement to traditional social-skill training. Moreover, virtual reality applications seem to be advantageous in terms of enhancing motivation for therapy. This result is particularly interesting because recent models of cognition suggest that motivation is a core component of the relationship between social functioning and cognition in schizophrenia (Barch, 2005).

A Spanish research team has developed and integrated a virtual reality program into social-skill training (Rus-Calafell et al., 2013). Their intervention is based on a brief cognitive-behavioral training in social-skills developed by the same team, in conjunction with the Soskitrain program, which consists of seven activities targeting seven behaviors: social perception, social information processing, responding and sending skills, affiliative skills, assertive communication, instrumental role skills, and conversational skills. The virtual reality program allows patients to practice social interactions with virtual avatars and promotes the gradual learning of the repertoire of social-skills – from more basic skills like facial emotion recognition to more complex ones such as holding a conversation. A case study that used this therapy showed a positive change in facial emotion recognition, social anxiety, and conversation time, along with improved interpersonal communication and assertiveness, and fewer negative symptoms (Rus-Calafell et al., 2012). The authors recently replicated their results in a pilot study of 12 persons with schizophrenia (Rus-Calafell et al., 2014).

However, programs that use virtual reality applications in schizophrenia often focus on social-skills and do not take specific processes of social cognition into account – as emotion recognition, understanding of others’ intentions or analysis of the social context – which are in fact essential to appropriate social functioning. We developed a program we call RC2S (cognitive remediation of social cognition in schizophrenia) to take this difficulty into account. RC2S is aimed at training social cognition processes in a realistic environment, in line with both the social cognition profile of the individual and his/her specific functional outcomes.

## RC2S: A Computer-Based Cognitive Remediation Program to Improve Social Cognition

RC2S was developed in France through collaboration between the Rehabilitation Department of Le Vinatier Hospital in Lyon (the authors of the present article) and SBT Company (headed by F. Tarpin-Bernard). This global remediation program was developed with two main goals in mind. The first was to stay close to difficulties encountered by patients in their daily social life. In order to achieve this point, we developed an individualized, flexible program that addresses both specific impairments in social cognitive processes and the objectives of each patient. The second goal, which follows from the fact that transferring skills or benefits acquired during cognitive remediation to daily life is hard, was to design a program based on data from clinical, psychological, and nursing interviews, and to make use of simulation technologies through collaboration with ITycom, a company specializing in virtual reality.

**Figure 2:**
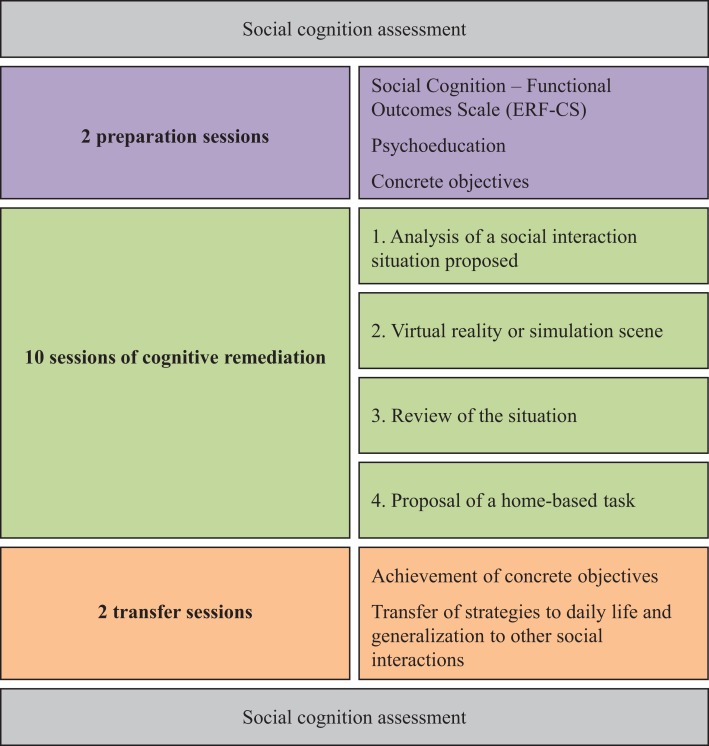
**Schematic representation of the RC2S therapy**.

Treatment with RC2S runs for 14 weeks, at a pace of one 1.5- to 2-h session per week (see Figure 2).

### Preparation sessions

The first two sessions are preparation sessions. In the first part of these sessions, the therapist and the patient look together at the social cognition assessment previously administered to the patient. The social cognition assessment battery used in RC2S (named ClaCoS) was developed by a French psychiatry research team made up of psychiatrists and neuropsychologists (GDR 3557). There is currently no consensus about what measures provide the best indicators of a given social cognitive domain, and the majority of existing social cognition measures have poor psychometric properties (Pinkham et al., 2013). To address these problems, the GDR team started from the social cognition psychometric evaluation (SCOPE) work (Pinkham et al., 2013) done in the USA, and sit out to identify the best existing French measures of social cognition – some of them are developed by the Versailles’ team, such as the LIS, which assess the understanding of implicit intentions by using an ecological video-based task (Bazin et al., 2009) – and develop others that did not exist in order to obtain a battery that would be suitable for assessing the domains of impaired social cognition in schizophrenia. Examining a patient’s social cognition assessment allows him/her to grasp his/her own profile, and to understand which of his/her own social cognitive components are impaired and which are preserved.

After this, the patient and his/her therapist investigate the functional outcomes of the impairments in the patient’s daily life. This investigation is made possible by a specific tool, the Social Cognition-Functional Outcomes Scale (ERF-CS, developed by E. Peyroux and B. Gaudelus)^1^. The ERF-CS is composed of 14 items that depict different social situations in which each component of social cognition is likely to have an impact. Items are ordered according to the main process involved. The scale provides an overall score of functional outcomes ranging from 0 to 154, and 4 subscores, one for each social cognition process.

The investigation of the patient’s objective difficulties (through the analysis of the social cognition assessment) along with the investigation of the repercussions of those difficulties on daily life, allow the patient to set two or three concrete objectives that should contribute to improving his/her social functioning. The last session of the preparation phase provides specific information about social cognition, impairments in schizophrenia or related disorders, and functional outcomes of these deficits, via a psychoeducational document (developed by E. Peyroux and B. Gaudelus, see text footnote 1). The aim of the psychoeducational session is to allow the patient to understand the specific terms used in the field of social cognition and increase motivation.

### Sessions of cognitive remediation

Then, 10 sessions of cognitive remediation are proposed, each composed of four parts: (1) an analysis of the social interaction situation proposed, (2) a virtual reality or simulation scene, (3) a review of the situation, and (4) a proposal of a home-based task. Each of the 10 sessions deals with a social interaction situation that is described in a text and simulated in the computerized program. The situations are ranked in increasing order of difficulty, based on both the emotional or affective nature of the situation and the complexity of the characters’ interactions. This allows us to abide by one of the fundamental principles of cognitive remediation: errorless learning, which requires to adapt difficulty of exercises to patient’s abilities, for the purpose of preventing implicit learning of errors.

#### Analysis of a social interaction situation

The first part of the cognitive remediation session consists of analyzing the proposed social interaction between Tom, the character interpreted by the patient during the therapy, and another character. No specific description of Tom is provided to patient to allow him/her to imagine Tom as close as possible of him/her. The interaction is described in a short text and the patient’s first task is to build a coherent mental representation of the situation by breaking it down into a microstructure. The patient has to be sensitive to the context and to take into account the links between his character, Tom, and the other character. In order to help him/her to decompose the situation, a “question wheel” is proposed. This part also allows the participant and the therapist to work on specific components of social cognition, such as Tom, by thinking about Tom’s or the other character’s mental states, intentions, or desires, and to find arguments supporting his/her choices. It also offers the opportunity to work on attributional bias by providing two possible outcomes for the situation: a positive one and a negative one. The patient has to explain the causes of the two outcomes based on external and internal attributions. Finally, some of the situations work on basic emotions (anger, fear, sadness, joy, etc.) by using photos and proposing to be more empathic with the different characters. Technologies used to build the program do not allow providing precise facial expressions according to each emotion. The focus in RC2S is more on body movements and emotional prosody. Therefore, RC2S may be associated with the use of other material as pictures or movies, when facial emotions have to be considered.

#### Simulation scene

After this first analysis step, the patient sits down in front of a computer to work on the social interaction in real-time. We chose to use a screen and not a particular immersion technique, firstly, in order to let patients experience a virtual environment that is less immersive because, as reported by Ku et al. (2006), such devices might be uncomfortable or difficult to control for people with schizophrenia, and secondly, immersion devices like CAVE (automatic virtual environment) and head mounted display (HMD) are relatively expensive, even if this point may be changing in the future. Insofar as our aim is to use RC2S as widely as possible, like other cognitive remediation techniques, we chose to use standard equipment that is already available in all hospital wards.

**Figure 3:**
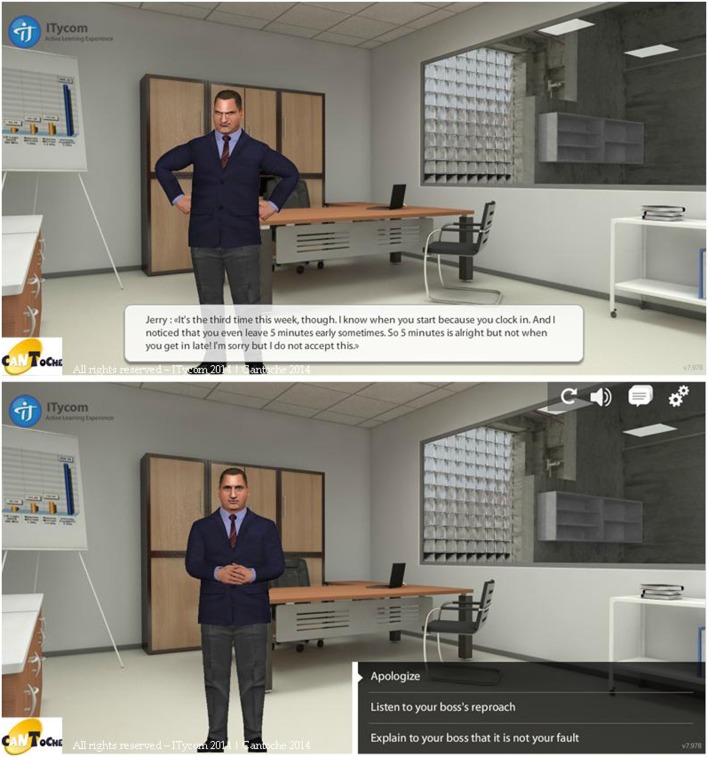
**Images from the RC2S program**. In this social interaction Tom who arrived at work late is reprimanded by his boss (top image, subtitles has been added). The patient is asked to select a direction for Tom’s behavior among those proposed (bottom image).

During the simulation scene, the patient’s goal is to assist Tom in the social situation and to guide him during the interaction by choosing a pattern of behavior (among those proposed) after each exchange between Tom and the characters (see Figure 3). To teach the ability to attribute intentions and mental states (Tom), and to analyze context (social perception), attitudes, prosody, and emotional dispositions (emotional processes), the patient is asked to select a direction for Tom’s behavior, but without any clear explanations of Tom’s speech. Moreover, during certain sequences, Tom’s behavior is predefined by the program. The patient thus needs to adapt to the evolving situation.

The social interaction scene follows a predefined but flexible decision tree where the patient’s choice influences Tom’s progress and upcoming interactions. Each of the 10 scenes lasts between 10 and 20 min. For each interaction, three types of behaviors are suggested based on models from several social-skill training or self-affirmation programs:

– Passive behavior: people fail to defend their rights, resolve difficulties by withdrawing, and express thoughts and feelings in an apologetic, self-effacing way. As a result, others easily disregard them.
– Aggressive behavior: people express their thoughts, feelings, and beliefs in a way that is usually inappropriate and always violates the rights of others. Difficulties are resolved by anger or aggressiveness.
– Assertive behavior: the person communicates his/her feelings, thoughts, and beliefs in an open, honest manner without violating the rights of others. Conflicts are resolved by negotiation.

In the RC2S program, the patient can browse through these three types of behavior in order to analyze the situation and also the interactions that follow.

In this step of the cognitive remediation session, the therapist does not intervene. The patient must verbalize the strategies employed to analyze the situation. The therapist has a caring attitude and should look for cues used by the patient to resolve the social interaction.

#### Review of the situation

The patient’s performance during the virtual reality scene is recorded. This makes it possible to decompose the patient’s behavior, interaction after interaction, which will be used in the third part of the cognitive remediation session: a review of the situation. The scene can be viewed as many times as desired and stopped at key times to allow the patient to take all of the social components (contextual information, tone of voice, gestures, and facial expressions) into account. This part helps the patient focus on specific components of social cognition. Depending on the impairments of each patient, the therapist can suggest that the patient analyze the characters’ gestures or facial expressions, listen to tones of voice in order to recognize emotion-specific inflections. The therapist can also ask the patient to do the scene over in order to generate specific mental states in the characters Tom is facing (for example, anger, envy, or discomfort), find key components to recognize in a real-time interaction, and to think about the characters’ possible reactions or intentions. This kind of adaptations linked to patients’ deficits can only take place through simulation technology, which enables one to come close to real interactions, test several kinds of behavior, and examine one’s interlocutor’s reactions without negative repercussions on daily life.

#### Home-based task

The cognitive remediation session finishes with a proposal of a home exercise. Home tasks are chosen by the patient in collaboration with the therapist in order to promote motivation. Exercises take into account both the level of performance of participant – tasks are thus of increasing difficulty to allow the patient to experiment consecutive successes – and related to the concrete objectives defined at the beginning of the therapy. Tasks are adapted to the needs of the patient and take into account his/her daily reality. For example, at the beginning of the therapy, the therapist can propose the patient to detect emotions and intentions of people in movies, and then in family context. Later, specific social interactions can be proposed to the patient and analyzed with the therapist. Finally, some tasks as the organization of activities with friends or family can be suggested. Home exercises are useful to promote the transfer of strategies to daily life and their subsequent automation. This is consistent with learning theories. Actually when cognitive remediation is provided in a rehabilitative context and reinforced in real-world settings, the learning process is facilitated and then generalization and transfer are promoted (Medalia and Saperstein, 2013). The home exercise is gone over with the therapist at the beginning of the next remediation session.

### Transfer sessions

Finally, two sessions are proposed at the end of the social cognitive remediation program. These sessions are transfer sessions. In these sessions, the therapist and the patient review the work done since the beginning of the therapy and look together at the achievement of the concrete objectives. These sessions also allow the patient to transfer skills acquired during the remediation program to his/her daily life by working close to his/her difficulties. Thank to these transfer sessions, the patient can adapt the strategies to other social interactions. To promote the reinforcement of strategies the patient set two or three new concrete objectives at the end of the therapy.

## Validation Methodology

The cognitive deficits experienced by patients with schizophrenia or related disorders are very diverse. Yet, most studies on the effectiveness of cognitive remediation treatment are randomized controlled trials. This approach has the advantage of minimizing allocation bias, balancing both known and unknown prognosis factors in the assignment of treatments and so reduces spurious causality. For example, in a very interesting randomized controlled trial from Wykes et al. (2007), symptoms of participants were rated by a psychiatrist unaware of group allocation, in a different building, and the study was carried out with blind randomization. This methodology also allows controlling focus on patients and increased social contact by using a control group similar to the therapy group in terms of focus, social contact, number, and duration of sessions. Moreover, randomized controlled trials can be combined in systematic reviews and are considered to be the most reliable form of scientific evidence. Nevertheless this methodology design also has some disadvantages because, depending on how heterogeneous the cognitive impairments are, group means cannot reflect the behavior of each person with schizophrenia (Shallice et al., 1991). Moreover, group studies have limited value when it comes to assessing the effectiveness of an intervention program for a given individual (Wilson, 2006). This is why single-case studies seem to be a better way to get as close as possible to the complexity of human social situations. Unfortunately, case study research is often misunderstood (for a summary of conventional wisdom regarding single-case studies see Flyvbjerg, 2006).

According to Tate et al. (2008), a single-case experimental design corresponds to “the intensive and prospective study of the individual, using an *a priori* methodology, which includes systematic observation, manipulation of variables, repeated measurements and data analysis.” In neurology, the single-case methodology is widely used to study the impact of cognitive remediation programs, but this is not the case in psychiatry. Some authors, however, have suggested using a single-case experimental design to assess individualized interventions involving functional issues (Kurtz et al., 2001). In line with the aims of the RC2S program, we have launched a validation study using multiple single-case experimental designs. We are currently testing the program with four patients: two people with schizophrenia, one patient with schizoid personality disorder, and one patient with 22q11 deletion syndrome. A thorough assessment, including a complete evaluation of components of social cognition and of social functioning, but also clinical and neuropsychological assessments has been proposed for each patient enrolled in the study. According to the patient’s profile and his/her objectives, we have collected three kinds of baselines before the beginning of the cognitive remediation intervention: baselines specific of the targeted component, non-specific baselines (such as measures of neurocognition processes that should not be affected by the intervention), and intermediary baselines that is measures of social cognitive function linked with targeted processes but not directly concerned by the cognitive remediation program. These measures and the complete assessment will be repeated at the end of the intervention to highlight impacts of RC2S on social cognitive impairments, and 6 months after to investigate a possible maintenance of the benefits. We don’t have yet objective results about the therapy; nevertheless, subjective reports from patients ongoing therapy are very positive. For the moment all the participants have been present in every sessions and are engaged in the therapy. They seem to develop social relationship by participating in activities outside their homes (such as sport, and activities with friends or relatives).

## Conclusion

As described in the first part of this paper, social cognitive treatments seem to be more effective on basic processes of social cognition like emotion recognition than on more complex or higher-order social cognitive functions, such as attributional style or social perception. According to Fiszdon and Reddy (2012) in order to impact higher-order processes, patients need to have ample opportunity for practice of skills until they become fully integrated and least somewhat automatic. We postulate that RC2S program, by using an environment close to the real social world that allows the patient to practice skills in specific social interactions, could have a greater impact on these complex social cognitive functions. To conclude, virtual reality or simulation techniques constitutes promising tools to study social cognition and treat patients with impairments in these areas, and so to favor rehabilitation in people with psychiatric troubles.

## Conflict of Interest Statement

The authors declare that the research was conducted in the absence of any commercial or financial relationships that could be construed as a potential conflict of interest.

## Footnotes

^1^https://wiki-afrc.org/afrc:documents
